# Screening and Identification of Reference Genes for *Paracarophenax alternatus*

**DOI:** 10.3390/insects17010007

**Published:** 2025-12-20

**Authors:** Yangming Zhang, Xu Chu, Ruiheng Lin, Yunfeng Zheng, Sikai Ke, Feiping Zhang, Songqing Wu

**Affiliations:** 1 Forestry College, Fujian Agriculture and Forestry University, Fuzhou 350002, China; 2 Key Laboratory of Integrated Pest Management in Ecological Forests, Fujian Agriculture and Forestry University, Fuzhou 350002, China

**Keywords:** *Paracarophenax alternatus*, pine wilt disease, *Monochamus alternatus*, reference genes, qRT-PCR

## Abstract

In this study, we focused on the screening and identification of reference genes for *Paracarophenax alternatus* Xu and Zhang. The laboratory population was used as the laboratory population, and samples were collected from mites at four different stages, including physogastry, viviparous, 5 d viviparous and phoresy. Then, the expression levels of seven candidate reference genes (*α-tubulin*, *β-tubulin*, *RPS18*, *RPL13*, *GAPDH*, *EF1A*, *SDHA*) were detected through qRT-PCR. Melting curves showed good gene specificity, and the amplification efficiency ranged from 90% to 102%. ΔCt analysis indicated that *GAPDH* was the most stable reference gene. The GeNorm software determined that the optimal number of reference genes was two, with *GAPDH* and *RPS18* forming the most stable combination, and NormFinder identified *RPS18* as the most stable reference gene. Although the BestKeeper software suggested that *EF1A* was the most stable, its *p*-value exceeded 0.05, rendering it unsuitable for use as a reference gene. Finally, through the RefFinder network tool, the most stable reference genes were identified as *GAPDH* and *RPS18*.

## Simple Summary

In this study, we focused on the screening and identification of reference genes for *Paracarophenax alternatus* Xu and Zhang, which is the primary natural enemy mite targeting the eggs of *Monochamus alternatus* Hope and has significant potential in the control of pine wilt disease. Samples were collected from mites at four different stages, including physogastry, viviparous, 5 d viviparous and phoresy, and the expression levels of seven candidate reference genes were detected through qRT-PCR. According to the ΔCt method, as well as the GeNorm, NormFinder, and BestKeeper software, *GAPDH* and *RPS18* were the most stable and represented the most suitable combination of reference genes among the four life stages of *P. alternatus*. This result lays the foundation for subsequent gene expression studies of this mite, which is believed to play a key role in host-seeking abilities, contributing to research on the biological control of pine wilt disease.

## 1. Introduction

Pine wilt disease (PWD) is a forest disease caused by the invasion of *Bursaphelenchus xylophilus* (pine wood nematode, PWN) and results in damage to pine trees [1]. PWD originated in North America and subsequently spread to regions such as China, Japan, South Korea, Spain, and Portugal, becoming a global biological threat [2,3]. PWD is characterized by its rapid spread, quick onset, high mortality rate, strong adaptability, and difficulty in controlling [4]. It is estimated that approximately 50% of the global pine distribution area will be affected by PWD infections in the future [5]. *Monochamus alternatus* Hope serves as the vector insect for PWD, and the control of *M. alternatus* populations is an important means to inhibit the spread of this disease. This can be achieved using natural enemies, which have the advantages of posing little harm to the environment and a low cost, offering a promising means to control vector insects [6]. For instance, *Phytoseiulus persimilis* Athias-Henriot has been utilized to manage *Tetranychus urticae* Koch in cucumber, strawberry, eggplant, and tomato crops [7]. Similarly, *Amblyseius swirskii* Athias-Henriot has been utilized to control *Thrips parvispinus* Karny and *Scirtothrips dorsalis* Hood in strawberry fields [8], and *Neoseiulus californicus* McGregor has been applied to control *T. urticae* in strawberry plantations in the United States [9]. These cases prove the practicality of using predatory mites to control pests, and it has been shown that they can penetrate smaller crevices to hunt their prey. *Paracarophenax alternatus* Xu and Zhang is a beneficial mite. This mite species undergoes four life stages: the physogastric phase, during which the female adult mite parasitizes the eggs of *M. alternatus*, feeding on the egg fluids to nourish and enlarge its abdomen for offspring development; the viviparous phase, referring to the first 12 h post-partum, during which viviparous mites are mature and capable of reproduction; the 5 d viviparous (senescent mite phase), characterized by heightened mortality and reduced activity as mites disperse in search of suitable hosts when none are readily available; and the phoresy phase, during which mites attach to the thorax and abdomen of *M. alternatus* for dispersal, entering a dormant state to extend their longevity until activated by the beetle’s oviposition, whereupon they migrate along the ovipositor to parasitize the eggs. One adult female mite can kill an egg and produce hundreds of offspring [10]. This species has the characteristics of a brief life cycle and robust reproductive capacity, making it a promising agent for biological defense [11]. However, the mechanisms of many physiological and biochemical processes in the life history of the mite remain unclear, and genetic studies are necessary to reveal the principles behind them; in this context, analyzing the quantitative expression of relevant genes is essential. Additionally, the screening and identification of reference genes for *P. alternatus* is key to understanding the mite.

Quantitative real-time PCR (qRT-PCR) boasts several advantages, including high sensitivity, stability, and reliability, as well as high efficiency and rapidity. This technique has become a staple in the field of molecular biology, playing an instrumental role in gene expression analysis [12]. The selection of reference genes is based on their high conservation and the assumption that they regulate the most fundamental life activities of cells. Ideally, these genes should be located in various parts of the organism and be stably expressed under general experimental treatments [13]. The stability of these genes is also relative. Studies have shown that differences in species and nutritional status, as well as environmental pressures, can affect the screening and identification of reference genes. As shown in Table 1, in quantitative gene expression studies, it is usually necessary to have two to three reference genes for mutual verification in order to ensure the accuracy of the results [14,15,16,17,18,19,20]. Based on the transcriptome results for *P. alternatus* and common reference genes for mites (see Table 1), seven candidate reference genes (*α-tubulin*, *β-tubulin*, *RPS18*, *RPL13*, *GAPDH*, *EF1A*, *SDHA*) were selected. After determining the candidate reference genes to be screened and the different life stages of the mites to be sampled, specific primer design was carried out. Samples were collected for RNA extraction and reverse transcription, and then qRT-PCR experiments were conducted to obtain the Ct values of the seven candidate reference genes across the four life stages. The most stable reference genes were selected based on four software programs: NormFinder (which can be used to simultaneously evaluate intragroup variation and intergroup variation and calculate stability values); BestKeeper (which allows evaluations of the stability of reference genes by calculating parameters such as the standard deviation (SD) and coefficient of variation (*p*)); GeNorm (which allows the optimal number of reference genes to be selected by calculating the stability of candidate genes’ expression compared to others and analyzing their variability through pairwise comparisons); and ΔCt (which is used to detect differences in the Ct values among all samples and compare the relative expression levels of the candidate reference genes across samples to assess their stability) [21]. Finally, the expression of the candidate reference genes across the four different life stages of *P. alternatus* was analyzed using the RefFinder network tool. The final output is a set of empirically validated and reliable internal standards, which can ensure the accuracy, reproducibility, and scientific validity of the results of subsequent research on quantitative gene expression [22].

## 2. Materials and Methods

### 2.1. Test Mites and Treatments

There may be differences in the stability of genes under different populations or environmental conditions. So, the specimen, *P. alternatus*, was obtained from the laboratory population at the Key Laboratory of Ecological Forest Pest Management, Fujian Province University. This mite was first discovered and named by Xu Yun and Zhang Feiping in 2010 in Minhou County, Fujian Province, China. Its adult form attaches to the ventral surface of the adult *M. alternatus* and spreads, and it has parasitic and lethal effects on the eggs of the *M. alternatus* [23]. The mite was bred and conserved on the eggs of *M. alternatus*, reared in the laboratory under a temperature of 26 °C, 90% RH, and total darkness [24]. Samples were collected in 1.5 mL centrifuge tubes across four life stages: 72 h physogastry, viviparous, 5 d viviparous, and phoresy (*M. alternatus* carried for 15 d). All samples were collected in 3 biological repeats with at least 30 mg per tube (about 1000 mites), after which they were snap-frozen in liquid nitrogen for 15 min and stored in a −80 °C refrigerator.

### 2.2. Total RNA Isolation and cDNA Synthesis

Total RNA was extracted from the collected mite samples using the HiPure Universal RNA Kit (Guangzhou Magen Biochemical Technology Co., Ltd., Guangzhou, Guangdong, China), following the kit instructions. The quality and concentration of the total RNA were examined using the NanoDrop^TM^ One Ultra-Micro Visible Spectrophotometer (Thermo Fisher Scientific Inc., Waltham, MA, USA), and the samples exhibited OD_260_/OD_280_ ratios ranging from 1.94 to 2.02. Subsequently, each sample underwent reverse transcription to synthesize the total cDNA, following the instructions for the Hifair^®^ III 1st Strand cDNA Synthesis Kit (Shanghai Yesen Biotechnology Co., Ltd., Shanghai, China). For the reverse transcription process, a 20 μL reaction system was used, comprising 4 × FQ-RT Super Mix.

The volume of total RNA was 5 μL; ddH_2_O was added to obtain a final volume of 20 μL, and the reverse transcription process was performed at 42 °C for 15 min (reverse transcription reaction) and 95 °C for 3 min (enzyme inactivation process). Total RNA was stored at −80 °C for cDNA synthesis, and total cDNA was stored at −20 °C for RT-qPCR.

### 2.3. Primer Design and Verification

Based on other reference genes related to mites, as well as transcriptome sequencing results, 7 commonly used reference genes were selected, i.e., *α-tubulin*, *β-tubulin*, *RPS18*, *RPL13*, *GAPDH*, *EF1A*, and *SDHA*. The sequences of the 7 reference genes were retrieved from NCBI (Table 2). Libraries were built locally, and the online NCBI tool primer-blast (https://www.ncbi.nlm.nih.gov/tools/primer-blast/ accessed on 5 June 2024) was used to design a quantitative PCR-specific primer (Table 2) with a length of 20 to 22 bases. The annealing temperature was 60 °C; the length of the amplified product was greater than 100 bp and less than 300 bp. The primers were synthesized by Fujian Shangya Biotechnology Co., Ltd., Fuzhou, Fujian, China.

### 2.4. RT-qPCR

A 20 μL reaction system was adopted, consisting of 10 μL Power Up SYBR Green Master Mix (Herui Biotechnolog Co., Ltd., Fujian, Fuzhou, China), positive and negative primers (2 μM) at 1 μL each, 1 μL cDNA template, and ddH_2_O as a supplement to reach 20 μL. qPCR was performed using the QuantStudio^TM^ 1 Plus system (Thermo Fisher Scientific Inc., Waltham, MA, USA) with a 3-step standard reaction pattern: UDG enzyme activation at 50 °C for 120 s; predenaturation at 95 °C for 300 s; denaturation at 95 °C for 10 s, annealing at 60 °C for 20 s, and extension at 72 °C for 20 s. A total of 40 cycles were carried out, and fluorescence signals were collected in the extension phase. Set the starting temperature of the dissolution curve to 60 °C and the ending temperature to 95 °C. Within this temperature range, read the fluorescence intensity data every 0.15 °C as the temperature increases. The dissolution curve was analyzed immediately after the qPCR reaction. For all four samples, 3 groups of biological replicates and 2 groups of technical replicates were included. At the same time, non-template control (NTC) or reverse transcriptase negative control should be set up to rule out genomic DNA contamination.

### 2.5. Standard Curve Construction and Verification of Primer Amplification Efficiency

The total cDNA template was subjected to gradient dilution, with each dilution being multiplied by five. This resulted in the acquisition of cDNA templates with dilution multiples of 1, 5, 25, 125, and 625. Subsequently, quantitative PCR (qPCR) was performed in accordance with the procedure outlined in Section 2.4. The results obtained were represented in a two-dimensional graph, with the log of the dilution of the cDNA template serving as the horizontal coordinate and the Ct value as the vertical coordinate. The statistical software SPSS 20 was employed to analyze the linear relationships between these variables, estimate the linear equation, and obtain the determination coefficient (R^2^) and slope. The amplification efficiency E was obtained according to the formula [14].

E = (10^(−1/slope)^ − 1) × 100

### 2.6. Data Processing and Analysis of Candidate Reference Genes’ Stability

Through the implementation of the ΔCt method and the GeNorm, NormFinder, and BestKeeper software, as well as the RefFinder online tool, the most stable candidate reference genes were identified.

The ΔCt method involves the use of raw Ct values obtained from RT-qPCR to analyze variations in Ct values across all samples, enabling a comparison of the relative expression levels of the candidate reference genes among different samples. It also enables the calculation of the standard deviation of the Ct values for each candidate reference gene within individual samples to determine suitable reference genes. However, the stability of the reference genes identified via the ΔCt method is determined relative to that of other genes, rather than being absolute. This approach primarily focuses on within-group variation and may overlook systematic between-group biases [25]. However, in this experiment, only four stages of the life cycle were involved, so there was no consideration of differences between groups.

GeNorm software calculates the average expression stability value (M-value) and enables the performance of pairwise comparisons to assess the influences of different factors. The gene with the lowest stability exhibits the highest M-value and is sequentially eliminated. A notable feature of this software is the ability to select two or more candidate reference genes, with the number of reference genes denoted by V. When the V_n_/V_n + 1_ ratio is less than 0.15, the number of reference genes corresponding to the smallest V_n_/V_n + 1_ ratio is adopted as the selection criterion [26]. Nevertheless, if two genes are co-regulated and exhibit simultaneous up- or downregulation, GeNorm software may still classify them as “stable”, but this in fact represents “co-instability”. Consequently, it may fail to detect genes with differential expression between groups.

Although NormFinder software operates based on similar computational principles to GeNorm software, aiming to determine the stability values of each reference gene, it differs in functionality by encompassing intersample variation calculations and the selection of a single optimal reference gene. If a reference gene shows significant differences between the control and treatment groups, NormFinder software assigns it a lower ranking, whereas GeNorm software might not detect such differences.

BestKeeper software is limited to comparing the expression levels of up to 10 reference genes with 10 target genes. Upon inputting Ct values, the software automatically computes parameters such as the standard deviation (SD) and Pearson correlation coefficient (*p*) to evaluate the stability of reference genes. A distinctive characteristic of BestKeeper software is its tendency to rate highly expressed genes (with low Ct values and small variations) as more stable. The software will sort the genetic stability based on the SD value. The genes with a smaller SD value have higher stability. However, it is particularly sensitive to outliers and can be heavily influenced by a single anomalous sample. The software recommends excluding genes with a standard deviation (SD) greater than 1, a criterion that may sometimes be overly stringent and lead to the exclusion of numerous genes. In order to ensure the completeness of the data analysis, this standard was not adopted.

Selecting a suitable set of reference genes requires the use of multiple algorithms for cross-validation, leveraging the strengths of each method and compensating for their weaknesses to ensure the highest possible stability of the selected reference genes, thereby guaranteeing the reliability of the subsequent quantitative results. Therefore, it is necessary to comprehensively analyze the results to select the most stable reference genes. RefFinder is an online tool (http://blooge.cn/RefFinder/ accessed on 5 June 2024) specifically designed for the screening and evaluation of the stability of reference genes. This tool enables the ranking of each gene by integrating different methods (such as GeNorm software, NormFinder software, BestKeeper software, and ΔCt methods) with weighting. The gene ranked 1 (the most stable) receives a weight score of 1, the gene ranked 2 receives a weight score of 2, and so on, with the gene ranked N receiving a weight score of N. For each candidate reference gene to be evaluated, it will receive four weight scores in the four algorithms. RefFinder calculates the geometric mean of these four weight scores for the gene. The smaller the geometric mean, the higher the ranking of the gene in each algorithm (i.e., the more stable). Finally, the genes are sorted from smallest to largest based on this geometric mean to obtain the final recommended ranking by RefFinder. The gene with the smallest geometric mean is recommended as the most stable reference gene.

## 3. Results

### 3.1. RNA Purity Determination and Quality Control

The RNA concentration and purity were determined using a micro-nucleic acid protein concentration analyzer. The results (Table 3) showed that the OD260/280 values of all extracted RNAs were between 2.07 and 2.12, and the OD260/230 values were between 2.25 and 2.36. This indicates that the RNA extracted from each developmental stage of *P. alternatus* is of good purity and can be used as a template for real-time fluorescence quantitative PCR reactions.

**Figure 1:**
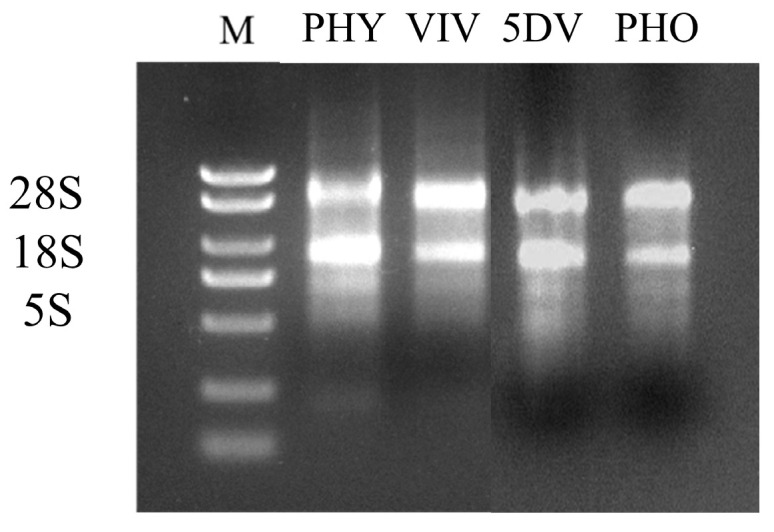
A: Electrophoresis of total RNA from different life stages of mites (PHY: 72 h physogastry; VIV: viviparous; 5DV: 5 d viviparous; PHO: phoresy, *M. alternatus* carried for 15 d).

The RNA was subjected to agarose gel electrophoresis for detection, and the results showed (Figure 1): The 28S and 18S bands were clear, without trailing bands, and the brightness ratio was approximately 2:1. The 5S band was relatively faint or almost undetectable, indicating that the RNA degradation was minimal and of good quality, which could meet the requirements of subsequent experiments.

### 3.2. Melting Curves, PCR Efficiency, and Product Specificity

**Figure 2:**
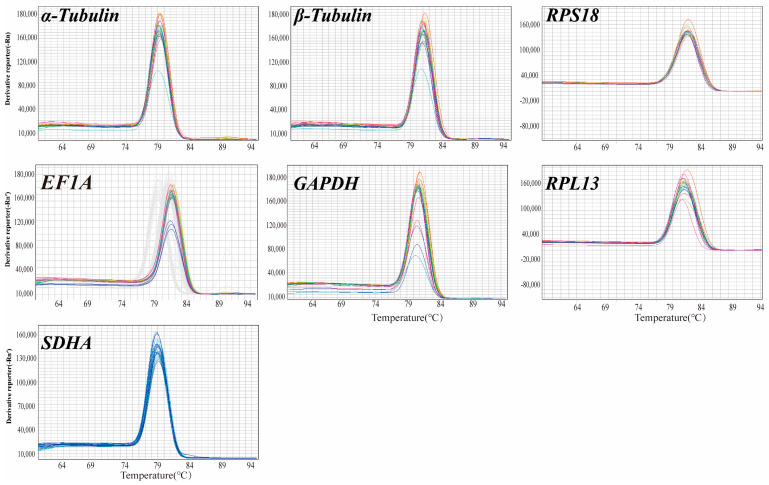
Melting curves of candidate reference genes.

The melting curve analysis achieved via qRT-PCR (Figure 2) demonstrated that the seven candidate reference genes (*α-tubulin*, *β-tubulin*, *RPS18*, *RPL13*, *GAPDH*, *EF1A*, and *SDHA*) exhibited no additional peaks beyond the primary peak, exhibiting excellent specificity. The amplification efficiency (E) was subsequently calculated and ranged from 90% to 102%, suggesting the efficacy of the RT-qPCR reaction (Table 1). Consequently, subsequent experiments could be conducted.

### 3.3. Analysis of Expression Stability of Candidate Reference Genes at Four Different Life Stages

**Figure 3:**
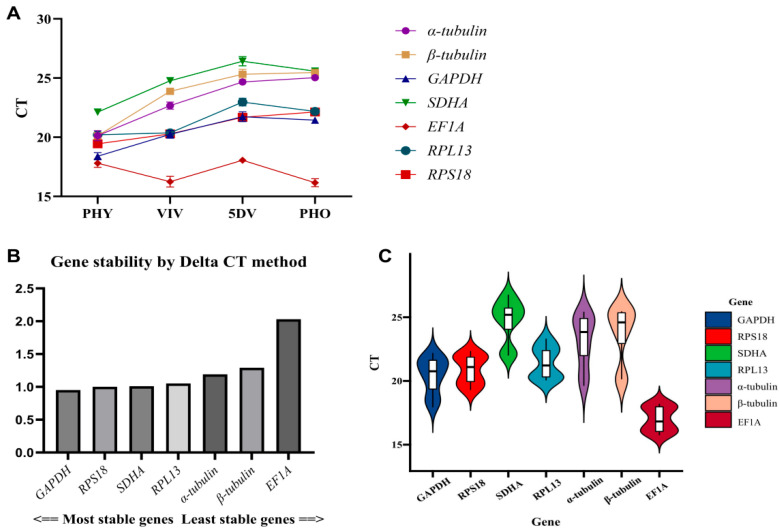
(**A**): Differences in reference gene expression are illustrated through line charts depicting the distribution of their cycle threshold (Ct) values. (**B**): Ct values of seven reference genes in all samples. (**C**): Gene stability according to ΔCt value evaluation.

In this study, qPCR was utilized to ascertain the expression levels of the seven candidate reference genes across four distinct stages of *P. alternatus*. The gene expression levels were subsequently plotted on a bar chart, shown in Figure 3A, and a boxplot, shown in Figure 3B. The stability of these genes was evaluated according to the ΔCt value (where a smaller ΔCt value indicates greater stability in gene expression).

The Ct values of the seven candidate reference genes ranged from 15 to 27, indicating sufficient gene expression abundance for the analysis of gene expression stability. *EF1A* was the reference gene with the highest abundance level, while *SDHA* had the lowest abundance (Figure 3A). Across the four life stages, the Ct value of *EF1A* exhibited the smallest range of fluctuation, with a difference of 2.114. This was followed by *RPL13* (3.032), *RPS18* (3.079), *GAPDH* (3.457), and *SDHA* (4.718) (Figure 3B). Moreover, the Ct value of α-tubulin exhibited the largest range of fluctuation, with a difference of 5.812. When evaluating stability based on ΔCt values, stable reference genes should have a constant expression level relative to one another. In other words, in any sample, the Ct values between two good reference genes should remain essentially unchanged. If their values fluctuate significantly, this indicates that at least one gene’s expression is unstable. Therefore, in the process of selecting reference genes, it is necessary to consider the overall pattern of changes in the candidate reference genes in different samples. Although the Ct value of *EF1A* exhibited the smallest range of fluctuation, this candidate reference gene showed increased expression in VIV, contrasting the expression trends of other genes, resulting in the largest difference in the ΔCt value. Therefore, it was not suitable for use as a reference gene. The candidate reference genes *RPL13* and *RPS18* were not down-regulated in VIV; they were slightly inferior to *GAPDH*. Thus, the final results of the ΔCt procedure yielded the following ranking regarding the stability of the candidate reference genes’ expression: *GAPDH* > *RPS18* > *SDHA* > *RPL13* > *EF1A* > *α-tubulin* > *β-tubulin* (Figure 3C). *GAPDH* is the most suitable reference gene for selection.

**Figure 4:**
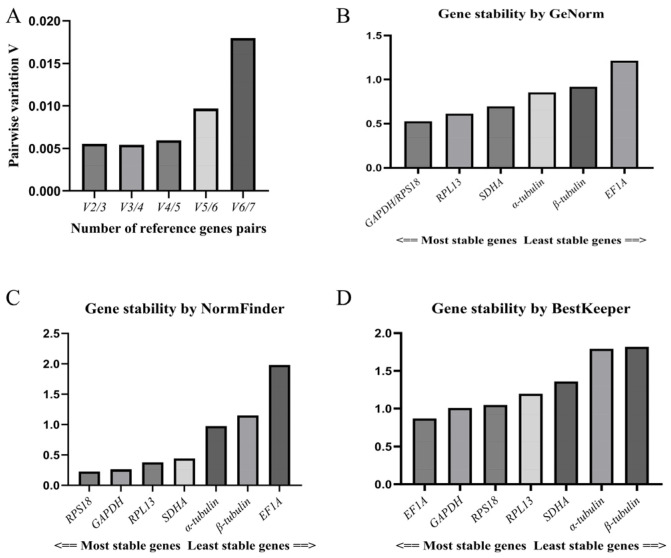
(**A**): Evaluation of number of candidate reference genes; (**B**): GeNorm software analysis of stability of candidate reference genes; (**C**): NormFinder software analysis of stability of candidate reference genes; (**D**): BestKeeper software analysis of stability of candidate reference genes.

The coefficient of variation analysis in the GeNorm software showed that the V_n_/V_n + 1_ values of the seven candidate reference genes were all less than 0.15 (V_2_/V_3_ = 0.005), and the minimum value taken to determine the number of candidate reference genes was 2 (Figure 4A). The stability of the candidate reference genes was ranked in descending order as follows: *GAPDH*/*RPS18* > *RPL13* > *SDHA* > *α-tubulin* > *β-tubulin* > *EF1A*. Thus, *GAPDH* and *RPS18* were the most stable reference genes according to this procedure, followed by *RPL13* and *SDHA*, while *EF1A* was the most unstable reference gene (Figure 4B). These are similar to the results obtained via the ΔCt procedure.

The results of the NormFinder software analysis were generally the same as those derived using the ΔCt method and GeNorm software, and the stability of the candidate reference genes was ranked in descending order as follows: *RPS18* > *GAPDH* > *SDHA* > *RPL13* > *α-tubulin* > *β-tubulin* > *EF1A*. The most stable gene was found to be *RPS18*, followed by *GAPDH*, *SDHA*, and *RPL13*, while the least stable gene was, again, *EF1A* (Figure 4C).

The BestKeeper software analysis yielded the following order regarding the expression stability of the candidate reference genes: *EF1A* > *GAPDH* > *RPS18* > *RPL13* > *SDHA* > *α-tubulin* > *β-tubulin* (Figure 4D). Although *EF1A* was shown to have the smallest range of Ct values (see Figure 3A,B), and the highest stability of *EF1A* was determined in the BestKeeper software analysis, it was upregulated in the VIV and PHY stages. This represents a major difference from the other candidate reference genes. Moreover, its *p*-value (0.963) was greater than 0.05 (Table 4), rendering the stability value insignificant, and it was not suitable for use as a reference gene.

## 4. Discussion

Quantitative real-time PCR (qRT-PCR) has emerged as a pivotal tool in gene expression analysis due to its exceptional sensitivity, specificity, accuracy, and reproducibility [19]. However, the reliability of its analytical outcomes is contingent upon the selection of appropriate reference genes, as the absence of these can significantly compromise the results’ credibility. The selection of reference genes constitutes a critical aspect of qRT-PCR analysis, as these genes may undergo alterations across different species and experimental treatments [26]. Reference genes must maintain stability under the specific experimental conditions required for the study. Instability in reference genes, potentially caused by human interference or an inability to accurately reflect the true expression changes in target genes, can adversely affect the accuracy of qRT-PCR analysis. Ideally, the reference genes should be widely distributed throughout the various parts of the organism, participate in the most fundamental life activities of the organism, and maintain stable expression under routine experimental conditions. In the literature, it has been demonstrated that borrowing reference genes from other species with similar relationships and incorporating the results of transcriptomics analyses allows a range of candidate reference genes to be determined [27].

This study employed ΔCt values, GeNorm software, NormFinder software, BestKeeper software, and the RefFinder network tool to analyze the expression stability of seven candidate reference genes across four life stages of *P. alternatus*, aiming to identify the most suitable reference genes. The findings underscore the critical importance of verifying the expression stability of reference genes across different life stages. Variations in the ranking results of reference genes across different software (ΔCt, GeNorm, NormFinder, and BestKeeper) primarily stem from differences in algorithmic characteristics and sensitivity. Despite these discrepancies, in this work, these analytical procedures enabled the identification of *GAPDH* and *RPS18* as the optimal combination for use as reference genes for *P. alternatus*.

In this study, *GAPDH* exhibited stable expression across all four life stages of *P. alternatus*, performing optimally in the ΔCt and GeNorm software analyses and ranking second in NormFinder software and BestKeeper software. *GAPDH*, which is a highly conserved enzyme in eukaryotes, plays a crucial role in the glycolytic pathway by catalyzing the conversion of glyceraldehyde-3-phosphate to 1,3-bisphosphoglycerate and generating NADH. This process is essential for energy metabolism in mites, as glycolysis provides ATP and NADH across all four life stages. *RPS18*, which ranked first in NormFinder and second in the ΔCt and GeNorm software analyses, likely owes its stability to its critical role in translation as a ribosomal protein. It participates in ribosome assembly, facilitating mRNA and tRNA binding to ensure accurate protein synthesis, which is vital for mite life activities [28]. The minimal impacts of *GAPDH* and *RPS18* across the different life stages of mites make them an excellent reference gene combination to normalize target gene expression.

Notably, *EF1A* ranked last in ΔCt, GeNorm software, and NormFinder software but first in BestKeeper software. This discrepancy arose because *EF1A* exhibited the highest gene expression among the seven candidate reference genes, with smaller Ct values and lower absolute variation (SD), leading to a bias in BestKeeper software’s algorithm towards its stability. The other three algorithms instead focus on comparing the relative expression levels of candidate reference genes across different samples. *EF1A* showed upregulated expression in VIV, which was inconsistent with the expression trends of the other six reference genes, and it exhibited excessive upregulation in PHO. Consequently, *EF1A* was deemed unsuitable for use as a reference gene in ΔCt, GeNorm software, and NormFinder software due to the significant differences in its relative expression levels across samples. *EF1A*’s core function involves acting during the elongation phase of mRNA translation and participating in the recognition and clearance of misfolded proteins through interactions with ribosomes and specific factors, serving as a component of the quality control system. The reason for *EF1A*’s upregulation in VIV remains unexplained and warrants further investigation regarding its mechanisms.

From the expression trends of the reference genes across the four mite life stages, the PHY stage represents a critical period for mite reproduction, characterized by intensified cell division and differentiation, increased energy consumption, and heightened protein synthesis demands, resulting in the highest expression levels of the reference genes. In the VIV stage, mites actively seek hosts, with lower energy consumption and protein synthesis requirements, yet this stage represents a peak in mite vitality and activity, with relatively high gene expression levels. The 5DV stage marks the end of the mite life cycle, characterized by severe energy depletion, a lack of additional energy supplementation, and significantly reduced vitality, resulting in the lowest gene expression levels. The PHO stage is a unique phase wherein mites aggregate on the ventral surfaces of *M. alternatus* adults, utilizing the beetle’s movement for dispersal. Mites can be transmitted to the vicinity of *M. alternatus* eggs through the oviposition behavior of female beetles, with a single mite capable of parasitizing and killing *M. alternatus* eggs while producing offspring [10]. During this stage, mites remain dormant, with relatively low gene expression levels. However, mites undergo a rejuvenation process during this phase, potentially facilitated by *EF1A*’s role in recognizing and clearing misfolded proteins, thereby enhancing their vitality. This may explain the more pronounced upregulation of *EF1A* during this stage.

As a member of the Eukaryota, Arthropoda, Arachnida, and Acari, *P. alternatus* was selected for the screening of its reference genes from closely related species. The results indicated that *GAPDH* and *RPS18* were stably expressed in almost all stages, making them ideal reference genes in the study of the different life stages of *P. alternatus*. In the context of contemporary gene expression level studies, *GAPDH* and *RPS18* are frequently utilized as reference genes in the order *Acarina* within the framework of biological studies. For instance, Augustine et al. conducted quantitative real-time polymerase chain reaction (qRT-PCR) validation on five candidate reference genes in *Polyphagotarsonemus latus* after treatment with insecticides and temperature stress. Their findings indicated that *RPS18* was the most stable reference gene [28]. Sun et al. identified RPS18 as a candidate reference gene for the sensitive and methaclyne pyrethroid-resistant strains of the *T. cinnabarinus* [18]. Conversely, Niu et al. demonstrated that *EF1A* and *GAPDH* were the most stable reference genes in strains of the citrus *P. citri* subjected to different pesticide selection pressures [20]. Zhang et al. evaluated 10 candidate reference genes in *Amphitetranychus viennensis* Zacher after subjecting it to four common insecticides. The results showed that, although each insecticide had a unique combination of reference genes, *α-tubulin*, *EF1A*, and *GAPDH* were the most stable reference genes under chemical attack [29]. In another study, Yang et al. evaluated the stability of candidate reference genes in *A. viennensis* under various environmental changes, including temperature, humidity, photoperiod, and host plant shifts. Their findings indicated that *GAPDH* was a suitable reference gene for humidity and host plant changes [30]. These promising results provide reference information for the selection of candidate reference genes for *P. alternatus*. Although *GAPDH* and *RPS18* were the most suitable reference genes for the *P*. *Alternatus* in this experiment and have often been selected as the most stable reference genes in previous studies, this does not mean that this result is applicable to all mites. For instance, in the case of *Varroa* mites, *GAPDH* was the least stable candidate reference gene among the three acaricide treatments; in the analysis of candidate reference genes for different developmental stages of *Eotetranychus sexmaculatus*, GAPDH ranked second to last. Therefore, in order to deal with different species or treatments, screening reference genes is a necessary task [16,31].

*P. alternatus*, as a highly promising parasitic mite, has demonstrated a remarkable capacity to regulate the population of the pine wilt disease vector *M. alternatus*. However, contemporary research on *P. alternatus* is predominantly centered on behavioral and applicative aspects, with strong potential for further in-depth exploration in the future. It is expected that the reference genes of *P. alternatus* will be broadly applicable. It should also be noted that the reference genes selected for this study are applicable only to the four different life stages of mites. It may not be universally applicable to other experimental treatments.

## 5. Conclusions

**Figure 5:**
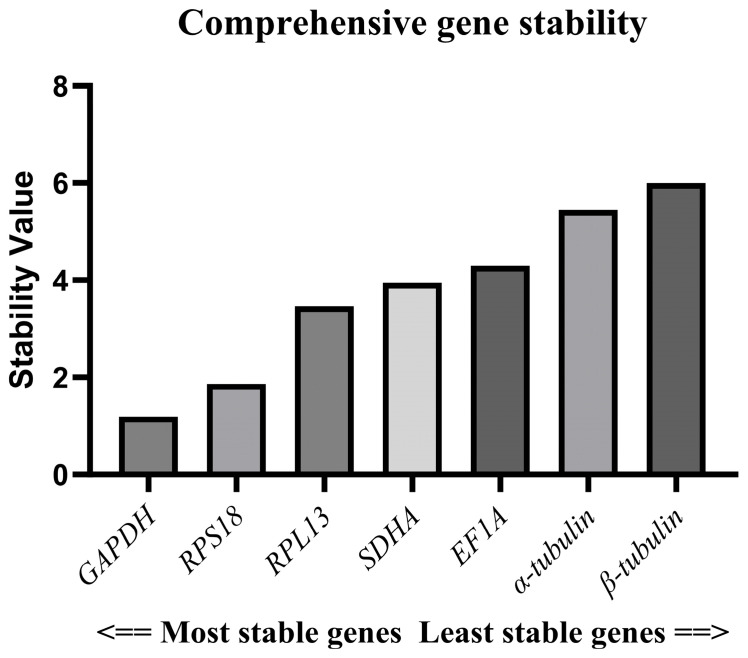
Assessment of reference genes’ stability according to RefFinder online tool.

The rankings of each gene according to the ΔCt method, GeNorm software, NormFinder software, and BestKeeper software were weighted and integrated through the RefFinder network tool. This tool operates by creating a comprehensive ranking, thereby eliminating the limitations of using a single method and enhancing the objectivity in the selection of internal references. Finally, comprehensive stability values were generated through the geometric mean or weighted average. The stability ranking of the seven candidate reference genes was as follows: *GAPDH* > *RPS18* > *RPL13* > *SDHA* > *EF1A* > *α-tubulin* > *β-tubulin* (Figure 5). Since the GeNorm software suggests that two reference genes should be selected as a reference gene pair, we selected the most stable reference genes, *GAPDH* and *RPS18*, as the optimal combination. This is also the most stable and suitable reference gene pair recommended in the GeNorm software.

## Figures and Tables

**Table 1:** Screening of reference genes for other types of mites.

Author	Title of Thesis	Condition	Optimal Reference Gene
Yue, L.X.; Gao, X.J.; Wang, J.J.; Lv, J.J.; Shen, H.M.	Selection of reference genes and study of the expression levels of detoxifying enzymes of *Tetranychus urticae*	Susceptible (SS) and resistant strains (Fe-R)	*α-Tubulin*, *SDHA* [15]
Liang, X.; Chen, Q.; Wu, C.L.; Fang, Y.J.	Selection of Reference Genes for Transcription Analysis of *Eotetranychus sexmaculatus* Superoxide Dismutase Gene EsSOD	Different life stages (i.e., larva, protonymph, deutonymph and female adults)	*α-Tubulin*, *β-Tubulin* [16]
Sun, W.; Jin, Y.; He, L.; Lu, W.C.; Li, M.	Suitable Reference Gene Selection for Different Strains and Developmental Stages of the Carmine Spider Mite, *Tetranychus cinnabarinus*, using Quantitative Real-Time PCR	Different developmental stages (eggs, protonymphs, nymphs, and adults)	*RPS18*, *α-Tubulin* [17]
Yang, C.X.; Pan, H.P.; Liu, Y.; Zhou, X.G.	Stably Expressed Housekeeping Genes across Developmental Stages in the Two-Spotted Spider Mite (*Tetranychus urticae*)	Different developmental stages (eggs, protonymphs, nymphs, and adults)	*RPL13*, *v-ATPase* [18]
Niu, J.Z.; Dou, W.; Ding, T.B.; Yang, L.L.; Shen, G.M.; Wang, J.J.	Evaluation of suitable reference genes for quantitative RT-PCR during development and abiotic stress in *Panonychus citri*	Various abiotic stresses (Thermo stress, UV irradiation stress, Acid rain stress, Acaricides stress)	*EF1A*, *GAPDH* [19]
Zhou, X.L.	Selection of the most suitable reference genes and expression profiling of CYP392A subfamily genes in the multi-pesticide resistant strain of *Tetranychus urticae*	The different developmental stages of the susceptible strain and multi-pesticide resistant strain (eggs, protonymphs, nymphs, and adults)	*EF1A* [20]

**Table 2:** Sequence, amplification efficiency and determination coefficient of the RT-qPCR primers for candidate reference genes.

Gene	Primer Sequences	Amplicon (bp)	PCR Efficiency	Slope	R^2^ Value
*α-tubulin*	F-GGTGGTGGTACTGGTTCAGG R-TCATTGTCAACCATGAAAGCACA	197	95.87%	−3.4246	99.4%
*β-tubulin*	F-CTGGGACGGTAAGTGCTCTG R-CCTGCTTACGTTTCCCTGGT	150	91.18%	−3.5531	99.85%
*RPS18*	F-ACAGTTCTTCCACGACGACC R-ACAAAAAGACCCAAAGGATGGT	180	101.59%	−3.2844	99.65%
*RPL13*	F-GCTTCCTTTGCTTTCTTTGCG R-ACCTTCCGCATTACGTGCTT	165	90.76%	−3.5650	99.14%
*GAPDH*	F-ATCGAAAACGCAGCTTGCAG R-GTCGTGGTGCAGGACAAAAC	296	90.13%	−3.5837	99.75%
*EF1A*	F-GCAACAATCAAGACAGCGCA R-GGCCGAAAGAGAACGTGGTA	158	92.56%	−3.5140	99.64%
*SDHA*	F-TGTTCCCCAAACGGCTTCTT R-GGAACACAGACCTGGTGGAG	254	90.65%	−3.5683	99.93%

**Table 3:** All RNA purity information.

Extracted RNA	OD_260/280_	OD_260/230_
Physogastric phase	2.12	2.25
Viviparous phase	2.10	2.32
5 d viviparous (senescent mite phase)	2.07	2.27
Phoresy phase	2.08	2.36

**Table 4:** Pearson correlation coefficient (r) and SD value.

BestKeeper vs.	*GAPDH*	*β-Tubulin*	*α-Tubulin*	*SDHA*	*EF1A*	*RPL13*	*RPS18*
coeff. of corr. [r]	0.973	0.950	0.987	0.963	0.015	0.934	0.954
*p*-value	0.001	0.001	0.001	0.001	0.963	0.001	0.001
SD	1.01	1.79	1.82	1.36	0.87	1.20	1.05

## Data Availability

All relevant data are included in the paper.
